# Risk of Myopathy in Patients in Therapy with Statins: Identification of Biological Markers in a Pilot Study

**DOI:** 10.3389/fphar.2017.00500

**Published:** 2017-07-27

**Authors:** Giulia M. Camerino, Olimpia Musumeci, Elena Conte, Kejla Musaraj, Adriano Fonzino, Emanuele Barca, Marco Marino, Carmelo Rodolico, Domenico Tricarico, Claudia Camerino, Maria R. Carratù, Jean-François Desaphy, Annamaria De Luca, Antonio Toscano, Sabata Pierno

**Affiliations:** ^1^Section of Pharmacology, Department of Pharmacy and Drug Sciences, University of Bari Aldo Moro Bari, Italy; ^2^Department of Clinical and Experimental Medicine, University of Messina Messina, Italy; ^3^Department of Biomedical Sciences and Human Oncology, University of Bari Aldo Moro Medical School Bari, Italy

**Keywords:** Skeletal muscle, statin, risk factor, chloride channel, lipid-lowering therapy, muscle biopsies, protein expression

## Abstract

Statin therapy may induce skeletal muscle damage ranging from myalgia to severe rhabdomyolysis. Our previous preclinical studies showed that statin treatment in rats involves the reduction of skeletal muscle ClC-1 chloride channel expression and related chloride conductance (gCl). An increase of the activity of protein kinase C theta (PKC theta) isoform, able to inactivate ClC-1, may contribute to destabilize sarcolemma excitability. These effects can be detrimental for muscle function leading to drug-induced myopathy. Our goal is to study the causes of statin-induced muscle side effects in patients at the aim to identify biological markers useful to prevent and counteract statin-induced muscle damage. We examined 10 patients, who experienced myalgia and hyper-CK-emia after starting statin therapy compared to 9 non-myopathic subjects not using lipid-lowering drugs. Western Blot (WB) analysis showed a 40% reduction of ClC-1 protein and increased expression of phosphorylated PKC in muscle biopsies of statin-treated patients with respect to untreated subjects, independently from their age and statin type. Real-time PCR analysis showed that despite reduction of the protein, the ClC-1 mRNA was not significantly changed, suggesting post-transcriptional modification. The mRNA expression of a series of genes was also evaluated. MuRF-1 was increased in accord with muscle atrophy, MEF-2, calcineurin (CN) and GLUT-4 transporter were reduced, suggesting altered transcription, alteration of glucose homeostasis and energy deficit. Accordingly, the phosphorylated form of AMPK, measured by WB, was increased, suggesting cytoprotective process activation. In parallel, mRNA expression of Notch-1, involved in muscle cell proliferation, was highly expressed in statin-treated patients, indicating active regeneration. Also, PGC-1-alpha and isocitrate-dehydrogenase increased expression together with increased activity of mitochondrial citrate-synthase, measured by spectrophotometric assay, suggests mitochondrial biogenesis. Thus, the reduction of ClC-1 protein and consequent sarcolemma hyperexcitability together with energy deficiency appear to be among the most important alterations to be associated with statin-related risk of myopathy in humans. Thus, it may be important to avoid statin treatment in pathologies characterized by energy deficit and chloride channel malfunction. This study validates the measure of ClC-1 expression as a reliable clinical test for assessing statin-dependent risk of myopathy.

## Introduction

Statin therapy is important for the prevention and treatment of cardiovascular diseases (CVD), which represent the primary cause of mortality worldwide. Many large scale multi-center trials have demonstrated the efficacy of these compounds in reducing cardiovascular event rates and in improving survival (Vaughan and Gotto, 2004). For this reason, statins are by far the most common first-line drugs because of their predictable clinical benefits in reducing low-density lipoprotein cholesterol. More recently, statins have been proposed in therapy as anti-inflammatory and anti-cancer. Yet, these drugs can produce a variety of skeletal muscle adverse reactions ranging from muscle pain to muscle cell damage and eventually leading to severe rhabdomyolysis (Vaughan and Gotto, 2004). Such effects can reduce compliance or require statins discontinuation, exposing the patients to life-threatening cardiovascular event. The mean age of statin users is nearly 60 years, but this treatment is also recommended for people older than 65 years of age, who are at increased risk of hypercholesterolemia and cardiovascular disease (Grundy, 2002). In clinical trials that included people older than 65 years, the safety profile and relative benefits of statin treatment have been reported to be similar to those in young adult people (Maji et al., 2013). However, a biological heterogeneity exists among old subjects and the impact of statin adverse effects may be amplified through different mechanisms (Sica and Gehr, 2002). Different authors have studied the mechanisms by which statins affect skeletal muscle function and although it is not still well defined, it is likely that a complex series of events may concur. Depletion of cholesterol in myofiber cell membranes may predispose to myopathy (Vaughan and Gotto, 2004). Also the depletion of intermediates in the cholesterol biosynthetic pathway, such as ubiquinone (coenzyme Q10), may have implications on the oxidative phosphorylation and energetic metabolism. However, it has been found that intramuscular levels of ubiquinone are not reduced by statins, thus ubiquinone depletion represents a less likely cause of myopathy with statins (Vaughan and Gotto, 2004). Inhibition of the activity of CYP3A4 or inhibition of glucuronidation by drugs or due to aging process can increase serum levels of statins, thus, elderly individuals receiving combination therapy should be carefully monitored for potential muscle-related side effects (Vaughan and Gotto, 2004). Other proposed mechanisms that may explain myopathy include interruption of isopentenylation of selenocysteine-tRNA by statins or reduction of small GTP-binding proteins such as Rho, Ras, and Rac, implicated in cell growth, which may be defective also in old subjects (Olson et al., 1995). More recently, a perturbation of muscle protein and carbohydrate metabolism due to FOXO alteration has been evidenced to explain the muscle weakness and fatigability reported in statin myopathic individuals, together with MuRF1 and atrogin increase (Mallinson et al., 2012). Our previous studies show that the skeletal muscle chloride channel ClC-1 is a target of statin side effects (Pierno et al., 2006, 2009). This channel stabilizes resting membrane potential and is important for the control of skeletal muscle function. Indeed, mutations of ClC-1 channel lead to Myotonia Congenita, a disabling disease characterized by sarcolemma hyperexcitability and a delay in muscle relaxation after contraction (Desaphy et al., 2013; Imbrici et al., 2016a). In rats, we have demonstrated that statin treatment reduces expression of this channel and destabilize sarcolemma excitability, which likely contributes to myopathy (Pierno et al., 2009). In parallel, the protein kinase C (PKC), the activation of which closes the channel and reduces the resting chloride conductance (gCl), is involved in the ClC-1 channel impairment due to statin action (Pierno et al., 2009; Camerino et al., 2014a). Statin-induced myopathy may be exacerbated in aged animals, in which gCl is already lower with respect to that of the adults (Pierno et al., 2014; Camerino et al., 2016). Additional parameters could be affected because calcium signaling and energetic pathway are changed in skeletal muscle of statin-treated rats (Liantonio et al., 2007; Camerino et al., 2011).

Here we performed a translational study focusing on the side effects of statin in skeletal muscle of patients in therapy reporting myalgia, to better understand the causes of damage also in consideration of the age of the patients. Based on our preclinical studies, we thought it might be important to evaluate whether ClC-1 channel is involved in the modification of skeletal muscle function, due to statin treatment, also in humans. The identification of a parameter that can be considered index of the risk of myopathy is crucial to avoid discontinuation of therapy. In this study, muscle biopsies of statin-treated patients were used to measure mRNA and protein expression of ClC-1 channel and of its known modulators. In addition, we explored other pathways involved in energy production important for cell survival as well as in muscle degradation and/or muscle regeneration/differentiation. To our knowledge there are few information on the causes of statin-related myopathy, so this study would allow clinical benefit in terms of helping in the prediction of statin-induced muscle damage as well as in the proposal of therapies able to prevent adverse effects in humans. This study is the first one showing the modification of ClC-1 expression in skeletal muscle of human subjects and paves the way for future in depth investigations. The results of this study may be useful to manage statin therapy. Indeed, statin therapy should be not recommended or should be limited in elderly patients and/or in those conditions characterized by ClC-1 channel malfunction (Myotonia Congenita, Myotonic Dystrophy, etc) (Imbrici et al., 2016b).

## Materials and Methods

### Subjects

The study was approved by our institutional University Ethic Committee (local ethics Committees) and conducted in accordance with the Declaration of Helsinki. Written informed consent was received from participants prior to inclusion in the study. Twenty-two individuals reporting myalgia were screened in the Department of Clinical and Experimental Medicine of the University of Messina, and ten subjects (50- to 77-years-old, 2 females and 8 males) with a history of continuous statin assumption (statin myopathic subjects) were deemed suitable for inclusion into the study. Patients reported myalgia fatigability and/or muscle weakness. A prevalence of simvastatin therapy was observed, but also pravastatin, and atorvastatin were represented. Duration of therapy was variable between 1 month and 6 years. No concurrent therapy with amiodarone, colchicine, or other potentially myotoxic drugs was noted; no patient performed muscle exercise. All patients denied muscle symptoms before starting statin therapy. Onset of symptoms varied from few months to several years after beginning of therapy. In all the patients, medical and neurological histories were collected, with particular regard to family history of neuromuscular symptoms. Thyroid function, immunological and rheumatologic parameters, lactic acid, and CK were tested. Neurological examination including muscle strength measurements and electromyography were performed. All patients underwent a diagnostic muscle biopsy. For control group we selected 9 subjects (40- to 69-years-old, males) with no history of statin use, who reported myalgia and/or exercise intolerance. In these subjects a suspected myopathy was ruled out after clinical and laboratory investigation including muscle biopsy. Exclusion criteria were: any neurologic, muscular, genetic, or other condition known to affect muscle function; electrolyte abnormalities; untreated hypothyroidism; use of any medications known to inhibit statin metabolism by cytochrome P450 3A4. Subject characteristics are presented in **Table 1**. The number of collected biopsies was limited because of the stringent inclusion characteristics and because only a few patients accepted to undergo to a biopsy. All the available biopsies were analyzed here.

**Table 1:** Physical and clinical characteristic of statin treated myopathic subjects analyzed in this study.

Subject examined	Sex/age (years)	Statin therapy	Dose (mg)	Duration of therapy (years)	Symptoms	CK UI/l	EMG	BM morphology
FG	M, 63	Pravastatin	40	4	Myalgia, cramps	350	Fibrillation potential	Atrophic fibers
GP	M, 54	Simvastatin	40	6	Myalgia, fatigability	2300	Normal	Increasing of Type II fibers
LMP	M, 60	Rosuvastatin	10	5	Myalgia, muscle weakness	400	Myopathic pattern	Fiber size Variability
MN	M, 57	Simvastatin +Ezetimibe	10/20	Several months	Myalgia	600	Myopathic pattern	Some fiber type grouping
MG	M, 71	Simvastatin	40	4	Myalgia	600	Normal	Fiber size variability
MM	F, 77	Simvastatin	40	3	Myalgia, muscle weakness	240	Myopathic pattern	Presence of some COX negative fibers
MC	M, 63	Simvastatin	40	2	Myalgia, easy fatigability	300–2000	Normal	Vacuoles with lipid storage
PS	M, 50	Simvastatin	20	6	Myalgia	167–800	Normal	Presence of some COX negative fibers
RN	M, 66	Fluvastatin	40	5	Myalgia, cramps	700–1700	Fibrillation potentials	Some fiber type grouping
VM	F, 64	Atorvastatin	40	1 month	Myalgia, exercise intolerance	600	Normal	Type II fiber atrophy

### EMG Recordings

Needle EMG (biceps brachii, first dorsal interosseus, rectus femoris and tibialis anterior muscles) and surface antidromic sensory (sural and ulnar nerves) and orthodromic motor (ulnar and tibial nerves) nerve conduction studies (NCS) were performed according to standard procedures. Compound motor action potential (CMAP) distal latencies, amplitudes, conduction velocities and F-wave latencies were recorded. Sensory nerve action potential (SNAP) distal latencies and amplitudes were also recorded. The presence or absence of abnormal spontaneous activity (SA) was noted, along with the type and distribution of SA (fibrillations, positive sharp waves, complex repetitive discharges, and myotonic discharges).

### Histological Analysis of Muscle Biopsies

All the patients, after informed consent underwent a muscle biopsy of Vastus lateralis (VL) muscle. Muscle biopsy was performed under local anesthesia and muscle tissue was immediately frozen in liquid nitrogen cooled isopenthane. Histological study included routine morphological staining (hematoxylin-eosin, Gomori’s trichrome, glycogen and lipid staining, Congo red for amyloid) and histochemical analysis for NADH-diaphorase, LDH, SDH, COX, PPL, MADA, ATPase.

### Biochemical Analysis

Mitochondrial enzymes were determined in muscle homogenates. To measure mitochondrial respiratory chain enzymes activities in skeletal muscle, 40 mg of tissue was homogenized in 9 volumes of 0.15 M KCl, 50 mM Tris–HCl, pH 7.4 (CPT medium), and centrifuged at 2500 *g* for 20 min at 4°C. The supernatant was used also for protein determination. About 1.5 × 106 cells were harvested and suspended in phosphate buffer saline pH 7, sonicated in ice for 10 s and protein was determined by Bradford assay. Activities of mitochondrial respiratory chain complexes were assessed spectrophotometrically. CoQ10 assessment in skeletal muscle was performed by HPLC as previously described (Barca et al., 2016).

### Muscle mRNA Extraction and Expression Quantification in Human Biopsies

Human section of muscle biopsies were snap frozen in liquid nitrogen soon after removal and stored at -80°C until use (Portaro et al., 2015). For each muscle sample, the total RNA was isolated by an RNeasy Fibrous Tissue Mini Kit (Qiagen C.N. 74704, Valencia) and quantified using a spectrophotometer (ND-1000 NanoDrop, Thermo Scientific, United States). The reverse transcription, amplification and purification of RNA was performed by OvationPicoSL WTA system V2 (NuGEN C.N. 3312-24 USA). To first strand cDNA synthesis, 50 ng of total RNA was added to 2 μl A1 first stand buffer mix solution (NuGEN C.N S01493) and incubated at 65°C for 2 min. Afterward, 2,5 μl A2 First strand buffer mix (NuGEN C.N S01494), 0,5 μl A3 First strand enzyme mix (NuGEN C.N S01495) were added and incubated at 4°C for 2 min, 25°C for 30 min, 42°C for 15 min, 70°C for 15 min and 4°C for 2 min. To second strand cDNA synthesis all tube were added 9.7 μl B1 solution second strand buffer mix (NuGEN C.N S01496) and 0.3 μl B2 second strand enzyme (NuGEN C.N S01377) and incubated at 4°C for 1 min, 25°C for 10 min, 50°C for 30 min, 80°C for 20 min and 4°C for 2 min. To purification of cDNA each reaction solution were added 32 μl of bead suspension Agencourt RNA clean XP Beads (NuGEN C.N S01307), mixed and incubated at room temperature for 10 min. Afterward, the tubes were transferred to the 96R Ring Magnet Plate (Bekman C.N. a001219). The pellet of beads were washed for 3 time with 200 μl of 70% ethanol, and incubated for air dry for 20 min. To amplification of DNA the pellet of beads were suspended with 50 μl C2 of SPIA buffer mix (NuGEN C.N S01498), 25 μl of C1 SPIA primer mix (NuGEN C.N S01497) and 25 μl of C3 SPIA enzyme mix (NuGEN C.N S01499) and incubated for 4°C for 1 min, 47°C for 75 min, 95°C for 5 min and 4°C for 2 min. The amplification solutions were transferred to the 96R Ring Magnet Plate and incubated for 5 min, afterward the pellet of bead were recuperated and purified with high pure PCR product purification kit (N.O. 11732668001 Roche) (Tricarico et al., 2008; Dinardo et al., 2012; Camerino et al., 2013).

Real-time PCR was performed in triplicate using the Applied Biosystems Real-time PCR 7500 Fast system (United States), MicroAmp Fast Optical 96-Well Reaction Plate 0.1 ml (Life Technologies C.N. 4346906) and MicroAmp Optical Adhesive Film (Life Technologies C.N. 4311971). Each reaction was carried in triplicate on a single plex reaction. The setup of reactions consisted of 10 ng cDNA, 0.5 ml of TaqMan-GeneExpression Assays (Life Technologies), 5 ml of TaqMan Universal PCR master mix No AmpErase UNG (2×) (Life Technologies C.N. 4324018) and Nuclease-Free Water not Diethylpyrocarbonate (DEPC-Treated) (Life Technologies C.N. AM9930) for a final volume of 10 ml. The following RT-TaqMan-PCR conditions were as follows: step 1: 95°C for 20 s, step 2: 95°C for 3 s and step 3: 60°C for 30 s; steps 2 and 3 were repeated 40 times. The results were compared with a relative standard curve obtained by five points of 1:4 serial dilutions. The mRNA expression of the genes was normalized to the best housekeeping gene beta-2 microglobulin (*2bg*) selected from beta-2 microglobulin (*2bg*), beta-actin (*Actinb*) and glyceraldehyde-3-phosphate dehydrogenase (*Gapdh*) by GeNorm software. TaqMan Hydrolysis primer and probe gene expression assays were ordered with the assay IDs reported in the **Table 2**, by Life Technologies. For genes that are poorly expressed, such as *Hdac5* and *Notch1*, a pre-amplification by TaqMan PreAmp Master Mix (Life Technologies C.N. 4391128) was made before the Real-Time PCR experiments. The set-up of pre-amplification consisted by 250 ng of reverse-transcription (in 12.5 ml volume), 25 ml of TaqMan PreAmp Master Mix (2×) and 12.5 ml of pool assay 0.2× (containing *Hdac5, Notch1* and *2bg*). The solution was incubated at 50°C for 2 min, 95°C for 10 min and for 40 cycles of 95°C for 15 s and 60°C for 1 min. The methods of gene expression analysis are the same as those previously used (Pierno et al., 2012; Camerino et al., 2016). A complete list of details for each gene is provided in **Table 2**. The RT-PCR experiments were performed in agreement with the MIQE guidelines for qPCR, as already published (Bustin et al., 2009). Real Time-PCR measures were performed in 5 biopsies of control subjects and 6 biopsies of statin treated subjects. The minor number of data reported in some cases is due to exclusion of the outlier samples by means of a specific software (Graphpad outlier calculator). Statistical analysis was performed using Student’s *t*-test.

**Table 2:** List of genes analyzed and quantified in human muscle biopsies by real-time PCR (Applied Biosystem).

Gene symbol	Gene Name	IDs Assay
*Clcn1*	Chloride channel, voltage-sensitive 1	Hs00892505_m1
*Prkca*	Protein kinase C, alpha	Hs00925193_m1
*Prkcq*	Protein kinase C, theta	Hs00989970_m1
*Mef2d*	Myocyte enhancer factor 2D	Hs00954735_m1
*Hdac5*	Histone deacetylase 5	Hs00608366_m1
*Ppp3ca*	Protein phosphatase 3, catalytic subunit, alpha isozyme	Hs00174223_m1
*Nfix*	Nuclear factor I/X (CCAAT-binding transcription factor)	Hs00231172_m1
*Ryr1*	Ryanodine receptor 1 (skeletal)	Hs00166991_m1
*Atp2a1*	ATPase, Ca++ transporting, cardiac muscle, fast twitch 1	Hs01092295_m1
*Tnnt3*	Troponin T type 3 (skeletal, fast)	Hs00952980_m1
*Pvalb*	Parvalbumin	Hs00161045_m1
*Trim63*	Tripartite motif containing 63, E3 ubiquitin protein ligase	Hs00261590_m1
*Myod1*	Myogenic differentiation 1	Hs00159528_m1
*Notch1*	Notch 1	Hs01062014_m1
*Ppargc1a*	Peroxisome proliferator-activated receptor gamma, coactivator 1a	Hs01016719_m1
*Idh3a*	Isocitrate dehydrogenase 3 (NAD+) alpha	Hs01051668_m1
*Prkaa1*	Protein kinase, AMP-activated, alpha 1 catalytic subunit	Hs01562315_m1
*Irs1*	Insulin receptor substrate 1	Hs00178563_m1
*Slc2a4*	Solute carrier family 2 (facilitated glucose transporter), member 4	Hs00168966_m1
*Actb*	Actin, beta	Hs99999903_m1
*Gapdh*	Glyceraldehyde-3-phosphate dehydrogenase	Hs99999905_m1
*B2m*	Beta-2-microglobulin	Hs99999907_m1

### Western Blot Analysis

PKCθ and pPKCθ proteins were isolated according to our previous study (Camerino et al., 2014a). ClC1 protein was isolated according to Papponen et al. (2005) with some modifications. Briefly, human biopsies were homogenized in ice cold buffer containing 20 mM Hepes (pH 7.4), 2 mM EDTA, 0.2 mM EGTA, 0.3 M sucrose, 0.2 mM phenylmethylsulfonyl fluoride and protease inhibitors. Homogenates were centrifuged at 7000 × *g* for 5 min at 4°C. The supernatant obtained was centrifuged at 50,000 × *g* for 1 h at 4°C and the pellet was solubilized in 20–30 μl of the same buffer. Protein concentration was quantified using Bradford protein assay kit (Bio-Rad). Forty micrograms of protein was separated on a 4–12% SDS–PAGE and transferred onto nitrocellulose membranes for 1 h at 200 mA (Semi-Dry transfer blot; Bio-Rad). Membranes were blocked for 2 h with Tris–HCl 0.2 M, NaCl 1.5 M, pH 7.4 buffer (TBS) containing 5% non-fat dry milk and 0.5% Tween-20, incubated overnight at 4°C with primary antibody. The following dilution of primary antibodies were used: rabbit anti-pPKCθ (Thr538) (Cell Signaling Antibodies, c.n. 9377) 1:1000; rabbit anti-PKCθ (Cell Signaling Antibodies c.n. 12206) 1:500; rabbit anti-Clc1 (MyBiosource, c.n. MBS714620) 1:200 with TBS containing 5% non-fat dry milk. After three washes with TBS containing 0.5% tween-20 (TTBS) membranes were incubated for 1 h with secondary antibody labeled with peroxidase (1:5000 anti rabbit IgG, Sigma–Aldrich). Membrane was then washed with TTBS, developed with a chemiluminescent substrate (Clarity Western ECL Substrate, Bio-Rad) and visualized on a Chemidoc imaging system (Bio-Rad). Densitometric analysis was performed using Image Lab software (Bio-Rad). The software allows the chemiluminescence detection of each experimental protein band to obtain the absolute signal intensity. The density volume was automatically adjusted by subtracting the local background. For each sample, the relative intensity was calculated by normalizing the intensity of β-Actin (diluted 1:300, rabbit Anti Actin, c.n. A2066, Sigma–Aldrich) protein band as reference standard. Protein extractions and immunoblots for the determination of AMPK/phosphorylated AMPK were carried out according to Shih et al. (2014). Briefly, biopsies were homogenized in ice cold buffer containing 20 mM Tris–HCl (pH 7.4 at 4°C), 2% SDS, 5 mM EDTA, 5 mM EGTA, 1 mM DTT, 100 mM NaF, 2 mM sodium vanadate, 0.5 mM phenylmethylsulfonyl fluoride, 10 mg/mL leupeptin and 10 mL/mL pepstatin. Homogenates were centrifuged at 1500 × *g* for 5 min at 4°C. The supernatant obtained was quantified using Bradford protein assay kit (Bio-Rad). Fifty micrograms of protein was separated on a 12% SDS–PAGE and transferred onto nitrocellulose membranes for 1 h at 150 mA (Semi-Dry transfer blot; Bio-Rad). Membranes were blocked for 2 h with Tris–HCl 0.2 M, NaCl 1.5 M, pH 7.4 buffer (TBS) containing 5% non-fat dry milk and 0.5% Tween-20, incubated overnight at 4°C with primary antibodies. The following dilution of primary antibodies were used: AMPK (rabbit polyclonal, Cell Signaling Technology) 1:1000, phosphorylated AMPK (rabbit polyclonal, Cell Signaling Technology) 1:500 with TBS containing 5% non-fat dry milk. After three washes with TBS containing 0.5% tween-20 (TTBS) membranes were incubated for 1 h with secondary antibody labeled with peroxidase (1:5000 anti rabbit IgG, Sigma–Aldrich). Membrane was then washed with TTBS, developed with a chemiluminescent substrate (Clarity Western ECL Substrate, Bio-Rad) and visualized on a Chemidoc imaging system (Bio-Rad). Densitometric analysis was performed using Image Lab software (Bio-Rad). The software allows the chemiluminescence detection of each experimental protein band to obtain the absolute signal intensity. The density volume was automatically adjusted by subtracting the local background.

### Statistics

All data are expressed as mean ± SEM. A comparison of means between treated and the related control group was evaluated by the unpaired Student’s *t*-test.

## Results

### Clinical Characteristics of Statin Statin-Treated Patients

Family history was unremarkable for all the patients. The statin users examined in this study reported different degrees of myalgia and fatigability. Fasciculation and cramps were also observed as well as difficulty in deambulation. Clinical features were characterized by muscle pain in all the patients and fatigability in 2 out of 10. Two patients showed moderate proximal muscle weakness at lower limbs. No patients reported episodes of dark urine. Clinical chemistry showed a variable increase of CK in all the statin treated patients (**Table 1**).

### Electromyographic Recordings in Statin-Treated Patients

On needle examination, a mean and median of 6 muscles were sampled (range 2–9). Two of 10 patients had fibrillations potentials at lower limbs. Three out of 10 showed a myopathic pattern with short duration, polyphasic motor unit potentials in the proximal muscles at lower limbs. In 50% of the patients neurophysiological study was normal (**Table 1**).

### Histological Analysis of Muscle Biopsies of Statin Users and Control Subjects

**FIGURE 1:**
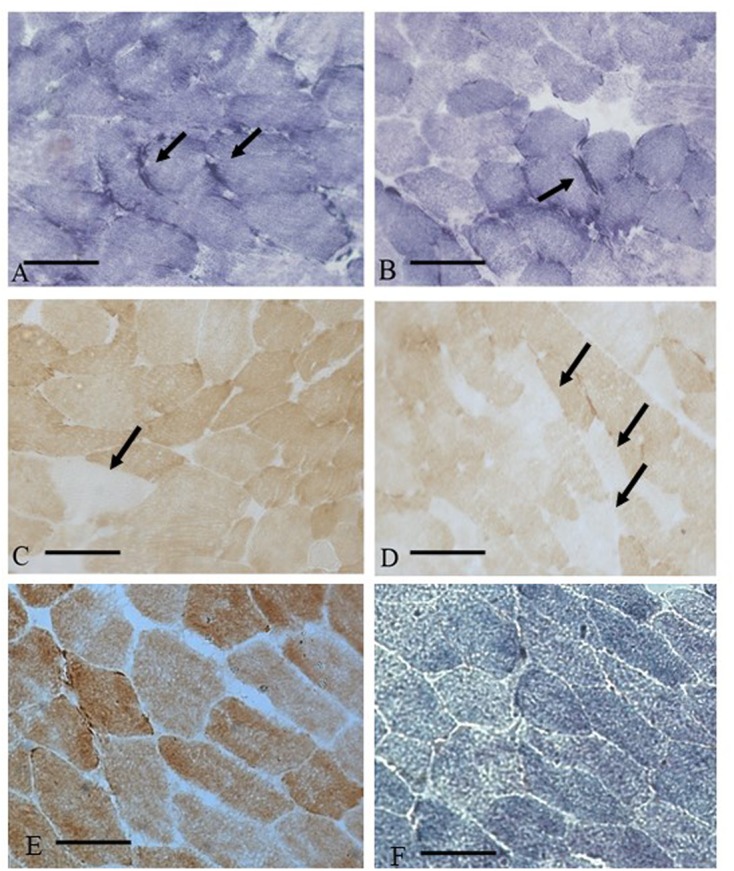
Muscle biopsy of a patient (MM) with statin myopathy showing SDH hyper-reactive and COX negative fibers. **(A,B)** 20× SDH staining showing muscle fibers with hyper-reactive sub-sarcolemmal rims (arrows). **(C,D)** COX staining with several COX negative fibers (arrows). **(E,F)** COX and SDH staining from a control subject.

In the statin-treated subjects, analysis of muscle tissue revealed a wide variety of histological changes ranging from minor morphological fiber alterations to mitochondrial and neurogenic alterations. The prominent morphological feature was fiber size variability associated with mitochondrial alterations (**Table 1**). **Figure 1** shows an example of skeletal muscle damage in a patient representative of the group in which modification was constantly observed. No alteration of muscle tissue was found in untreated subjects.

### Muscle Biochemistry

**FIGURE 2:**
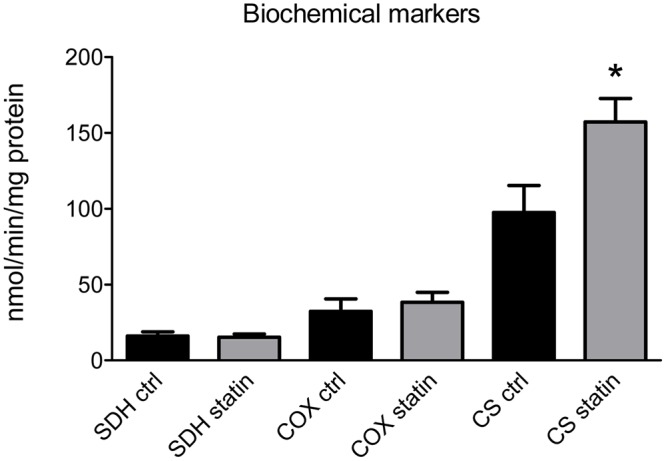
Activity of mitochondrial enzymes as a biochemical markers was measured in muscle biopsies of statin-treated myopathic (statin) and untreated (ctrl) subjects. Bars represent the mean value ± SEM of succinate dehydrogenase (SDH), cytochrome oxidase (COX) and citrate synthetase (CS) activity measured in 10 statin-treated subjects and 9 untreated subjects. ^∗^Significantly different with respect to control value (*p* < 0.05) by Student’s *t*-test.

Biochemical spectrophotometric analysis showed a significant increase of citrate synthase (CS) activity in statin-treated subjects (**Figure 2**), while succinate dehydrogenase (SDH) or cytochrome oxidase (COX) activities were unchanged (**Figure 2**). Also, no significant difference in CoQ10 level, evaluated by HPLC, was found in muscle biopsies between statin-treated and control subjects, being 22.4 ± 2.2 μg/gr tissue (*n* = 10) to 25.0 ± 4.0 μg/gr tissue (*n* = 9) (*P*-value = 0.5; n.s. by Student’s *t*-test).

### Muscle mRNA and Protein Expression of ClC-1 Chloride Channel, Protein Kinase C and Related Transcription Factors in Vastus Lateralis (VL) Muscle Biopsies of Statin-Treated Patients Showing Sign of Myopathy

**FIGURE 3:**
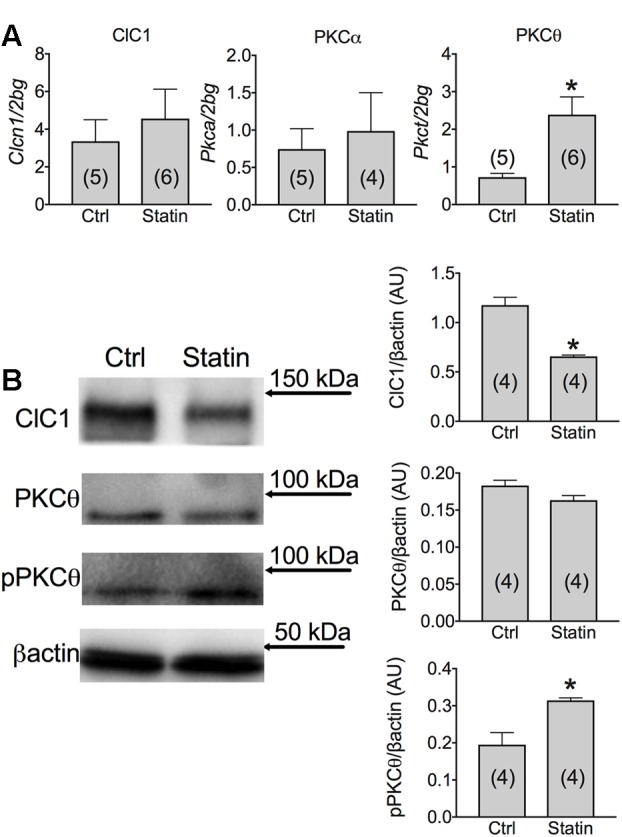
**(A)** Real-time PCR analysis of mRNA expression level of ClC-1 chloride channel and of PKCα and PKCθ in VL muscle biopsies of patients in therapy with statin showing sign of myopathy. Histograms show quantification of transcript levels normalized by the beta-2 microglobulin gene. Mean values ± SEM in myopathic subjects (Statin) were compared with those of untreated subjects (Ctrl). ^∗^Significantly different with respect to the own control (*P* < 0.05 or less) by Student’s *t*-test. **(B)** Representative Western blot showing the expression levels of ClC-1 and PKCθ proteins in the unphosphorylated and phosphorylated form in muscle tissues. The blots were reacted with specific antibodies. β-actin was used to normalize the blot. The densitometric analysis of each experimental band was performed using ImageLab software. Histograms show quantification of relative protein levels. Relative intensity was calculated by normalization of the absolute intensity of target protein with the absolute intensity of β-actin, as reference standard, and are represented as arbitrary units (AU). Each bar represents the mean ± SEM from the number of muscle biopsies as indicated. ^∗^Significantly different with respect to Ctrl (at least *P* < 0.05) by Student’s *t*-test.

The mRNA and protein expression of ClC-1 were measured in muscles biopsies of patients reporting myalgia, muscle cramps or hyper-CK-emia associated to statin treatment. We found that although the mRNA amount of ClC-1 channel was similar between the two groups, the expression of the ClC-1 protein was significantly decreased by 41.0 ± 1.8% in muscle biopsies of patients in therapy with statin (**Figure 3**). Such a reduction likely induce sarcolemma electrical instability which may contribute to muscle cramps, fasciculation and myopathy observed during statin therapy.

Importantly, a low variability was found between samples within each group. Despite the low number of biopsies analyzed, the effect size of ClC-1 protein expression was 4,572 and the statistic power (1-β) was 0.9995 (calculated using G-power software). Thus, it appears that variability in parameters, such as age, gender, and type of statin, has little influence on the results, being the modification of ClC-1 independent from them. It is worth noting that a reduction of less than 50% of ClC-1 channel activity is not expected to produce myotonia symptoms (Pusch, 2002), but may contribute to milder symptoms. We previously demonstrated that ClC-1 channel is modulated by the PKC in rodents (Pierno et al., 2007; Camerino et al., 2014a, 2015). In humans, we observed a significant increase of PKCθ mRNA in muscle biopsies of statin-treated myopathic patients with respect to the untreated subjects (**Figure 3**). In contrast, the PKCα was less affected in statin-treated patients, with only a slight increase of the mean value (**Figure 3**). Interestingly, the expression of total PKCθ protein was only slightly modified, while the proportion of the phosphorylated form was significantly increased, suggesting enhanced activity (**Figure 3**).

### Muscle mRNA Expression Level of Phenotype- and Calcium Homeostasis-Related Genes in Human Biopsies of Statin-Treated Patients Showing Sign of Myopathy

**FIGURE 4:**
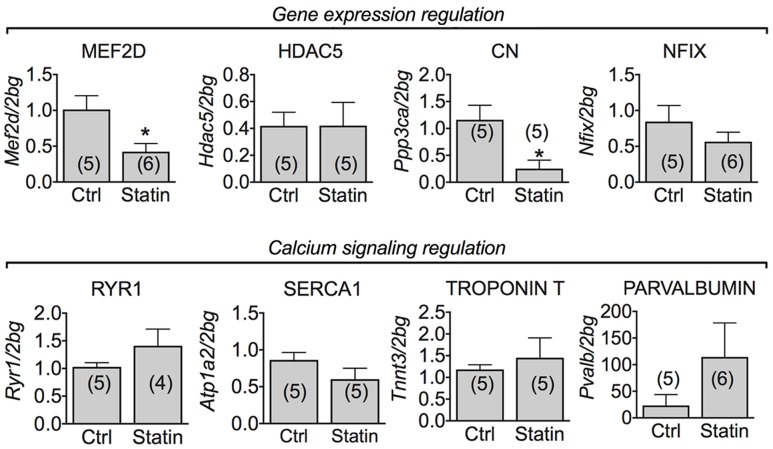
Real-time PCR analysis of mRNA expression level of genes involved in ClC-1 chloride channel regulation and calcium signaling in VL muscle biopsies of patients in therapy with statin showing sign of myopathy. Histograms show quantification of transcript levels for MEF2D, HDAC5, CN, NFix, RyR1, SERCA1, Troponin T and Parvalbumin normalized by the beta-2 microglobulin gene. In each graph, the bars represent the mean values ± SEM in myopathic subjects (Statin) compared with those of untreated subjects (Ctrl) from the number of subjects indicated in brackets. ^∗^Significantly different with respect to the own control (*P* < 0.05 or less) by Student’s *t*-test.

Calcineurin (CN), a phosphatase controlling muscle growth and slow-fiber type determination, through the modulation of the transcription factor MEF-2 (Hudson and Price, 2013), is also involved in the expression of ClC-1 channel in rats (Camerino et al., 2014a, 2015). In human biopsies, we found a significant reduction of mRNA encoding MEF-2 and CN. However, the mRNA expression of HDAC5 and Nfix, both involved in transcriptional regulation with MEF-2, was unchanged (**Figure 4**). To verify possible change of muscle phenotype due to statin treatment in the human biopsies we measured the troponin T3 transcript in the human biopsies, which is typically expressed in fast-twitch muscles. Troponin T3 mRNA expression was not modified in the biopsies of myopathic subjects (**Figure 4**). Also parvalbumin, RyR1 and SERCA1 gene expression was unaffected by statin treatment (**Figure 4**).

### Muscle mRNA Expression Level of Genes Involved in Degeneration and Regeneration Processes in Human Biopsies of Statin-Treated Patients Showing Sign of Myopathy

**FIGURE 5:**
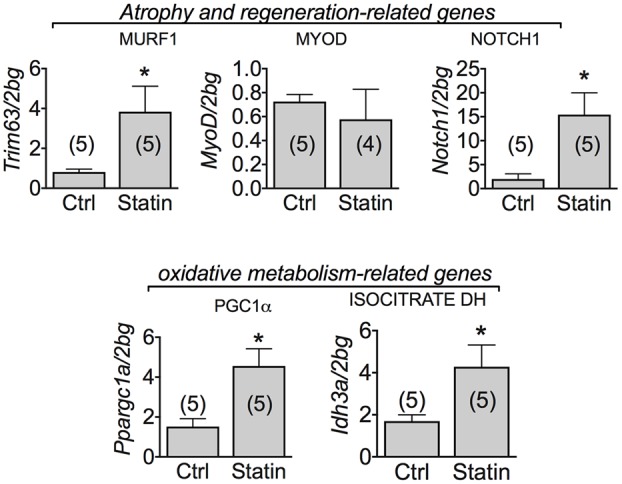
Real-time PCR analysis of mRNA expression level of genes involved in muscle atrophy and oxidative-metabolism related genes in VL muscle biopsies of patients in therapy with statin showing sign of myopathy. Histograms show quantification of transcript levels for MuRF1, MyoD, Notch1, PGC1α and IDH normalized by the beta-2 microglobulin gene. In each graph, the bars represent the mean values ± SEM in myopathic subjects (Statin) compared with those of untreated subjects (Ctrl) from the number of subjects indicated in brackets. ^∗^Significantly different with respect to the own control (*P* < 0.05 or less) by Student’s *t*-test.

**FIGURE 6:**
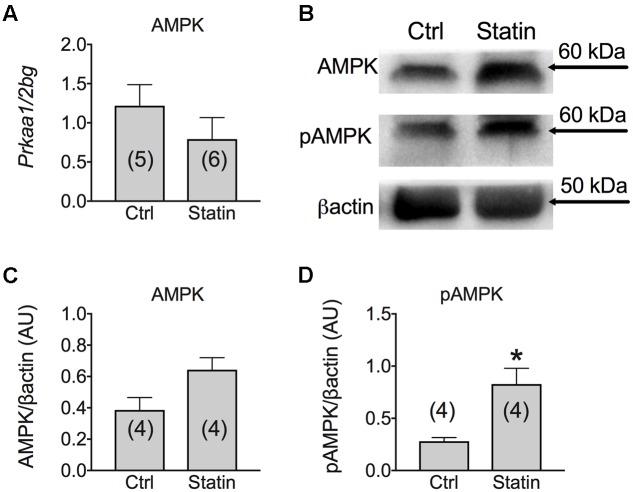
**(A)** Real-time PCR analysis of mRNA expression level of AMP Kinase (AMPK) gene involved in muscle energetic metabolism in VL muscle biopsies of patients in therapy with statin showing sign of myopathy. Histograms show quantification of transcript levels for AMPK normalized by the beta-2 microglobulin gene. In each graph, the bars represent the mean values ± SEM in myopathic subjects (Statin) compared with those of untreated subjects (Ctrl) from the number of subjects indicated in brackets. **(B)** Representative Western blot showing the expression level of total AMPK protein and its phosphorylated form in muscle tissues. The blots were reacted with specific antibodies. β-actin was used to normalize the blot. Histograms show quantification of relative protein levels of total AMPK **(C)** and pAMPK **(D)** obtained by densitometric analysis of each protein normalized with β-actin, as reference standard, and represented as AU. Each bar represents the mean ± SEM from the number of muscle biopsies as indicated. ^∗^Significantly different with respect to the own control (*P* < 0.05) by Student’s *t*-test.

According to previous studies (Hanai et al., 2007), we found that MuRF-1 was significantly increased in human biopsies of patients undergoing statin-related myopathy, indicating the ability of statin to stimulate proteolysis. We also examined the role of statin in regeneration processes by analyzing the expression of genes such as Notch-1, involved in the pathways of muscle regeneration and myoblast survival, and MyoD, involved in the differentiation process. We found a significant increase of Notch-1 in statin-treated myopathic patients with respect to control, while MyoD was unchanged (**Figure 5**). This effect suggests that, likely due to statin-induced muscle injury, satellite cells proliferate (Conboy and Rando, 2005) in an attempt to repair damaged tissue. Concomitantly, the mRNA level of PGC-1α was significantly increased, suggesting activation of mitochondrial biogenesis. Accordingly, isocitrate dehydrogenase 3, a mitochondrial enzyme, was significantly increased in subjects treated with statin and affected by myopathy (**Figure 5**). The measure of the AMP-activated kinase (AMPK) mRNA and protein expression, was only slightly modified after statin treatment (**Figure 6**). However, a significant increase of the phosphorylated and active form of AMPK was observed, suggesting the activation of cytoprotective pathways in response to energetic deficit (**Figure 6**).

### Muscle mRNA Expression Level of Glucose Metabolism-Related Genes in Human Biopsies of Statin-Treated Patients Showing Sign of Myopathy

**FIGURE 7:**
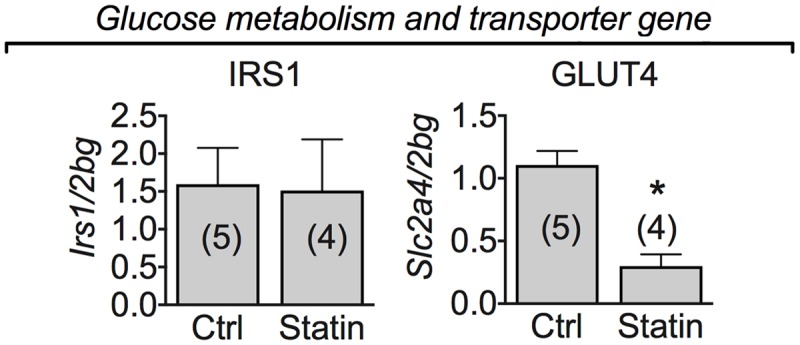
Real-time PCR analysis of mRNA expression level of genes involved in glucose metabolism and transporter genes in VL muscle biopsies of patients in therapy with statin showing sign of myopathy. Histograms show quantification of transcript levels for IRS1, and GLUT-4 normalized by the beta-2 microglobulin gene. In each graph, the bars represent the mean values ± SEM in myopathic subjects (Statin) compared with those of untreated subjects (Ctrl) from the number of subjects indicated in brackets. ^∗^Significantly different with respect to the own control (*P* < 0.05) by Student’s *t*-test.

Interestingly, the mRNA expression of glucose transporter type 4 (GLUT-4) was significantly decreased in all the patients examined reporting statin-induced myopathy (**Figure 7**). To verify whether this reduction was accompanied by the involvement of insulin signaling pathway we analyzed the mRNA expression of IRS-1, a signaling protein downstream the insulin receptor involved in metabolic pathways. Transcript for IRS-1 was not modified in statin-treated subjects with respect to controls. This suggests a reduced muscle capacity of glucose uptake due to GLUT-4 reduced mRNA expression.

## Discussion

The present study allowed the identification of molecular markers involved in the statin-induced myopathy in humans, which may help to classify individuals at higher risk. We evaluated the modification of gene and protein expression in skeletal muscle biopsies of patients in statin therapy and undergoing adverse muscle symptoms. We demonstrated that the ClC-1 channel is a key parameter in determination of statin-induced muscle side effects. In parallel, a biochemical and functional analysis has revealed significant correspondence with the molecular results and allowed new considerations important for therapy. This is the first evidence of the involvement of ClC-1 channel in the myopathy due to statin therapy in humans. The fundamental role of the ClC-1 channel has been established in congenital diseases such as Myotonia Congenita (MC) in which loss-of-function mutations in the ClC-1 gene determines sarcolemma hyperexcitability and failure of relaxation after contraction (Koch et al., 1992; Desaphy et al., 2013). Similarly, in patients undergoing statin therapy, the reduction of ClC-1 channel and of the related chloride conductance (gCl) may be critical for determining muscle side effects. The ClC-1 channel protein is markedly reduced in those patients showing the clinical signs of statin-induced myopathy, and in most of the cases, this effect occurs in parallel with a modification of the electromyographic recordings, although normal electromyograms do not exclude the presence of statin-induced myotoxicity (Taha et al., 2014). Being this channel important for sarcolemma electrical stabilization, the resulting hyperexcitability may likely contribute to cramps and myalgia. Differently from the results obtained in statin-treated rats, in which the ClC-1 transcript was reduced in accord with the reduction of ClC-1 protein and gCl (Pierno et al., 2009; Camerino et al., 2016), here the corresponding mRNA level was not significantly changed in muscle biopsies of statin-treated patients. We thus hypothesize an alteration of the translation or a rapid degradation of the protein due to activation of proteolytic systems (Chen et al., 2015). In addition, the activity of the channel may be further reduced due to the increased expression and activation of PKC, a negative modulator of ClC-1 activity. It is known that activation of PKC occurs during the onset of myofiber action potential firing, and, through reduction of the resting gCl, contributes to maintain myofiber excitability (Pedersen et al., 2016). In muscle biopsies of the statin-treated subjects, PKCθ transcript was significantly increased, thus appearing as a specific target of statin action. Additionally, the phosphorylated, active form of PKCθ was markedly increased, strengthening a negative regulation on the ClC-1 channel. It has been shown that myocyte enhancer factor-2 (MEF-2) and calcineurin (CN) are associated to ClC-1 expression and can modulate muscle phenotype determination (Wu and Olson, 2002; Pierno et al., 2013; Camerino et al., 2015). Indeed, CN activation promotes slow muscle genetic program through the increase of its molecular target MEF-2 (Wu et al., 2001) and in slow muscles the expression of ClC-1 is lower with respect to the fast-one. During inactivity, slow-twitch myofibers undergo to a slow-to-fast transition related to increase of ClC-1 mRNA, decrease of MEF-2 and muscle atrophy (Pierno et al., 2007, 2013). In biopsies of statin-treated subjects, a reduction of CN and MEF-2 mRNA expression was found, suggesting that statin can also affect their expression. This may suggest a shift toward a faster muscle phenotype (however, not compatible with mitochondrial biogenesis), but the expression of genes marker of fast-phenotype such as troponin T3, parvalbumin and SERCA were not significantly modified. Also, the nuclear factor one X (Nfix) gene, involved in the developmental transcription of a number of fetal-specific genes and in MEF-2 activation, was unchanged (Messina et al., 2010). Since MEF-2 and CN have been proposed as regulator of muscle growth (Wu and Olson, 2002; Hudson and Price, 2013), their decreased expression may be also associated to the MuRF-1 induced atrophy as well as to the reduction of transcription of specific muscle genes (Naya and Olson, 1999; Wu et al., 2001) such as the glucose transporter GLUT-4, which control glucose uptake and ATP production capacity. In addition, the possible statin-induced increase of the intracellular calcium in human myofibers, as shown in the rat (Liantonio et al., 2007) and human (Sirvent et al., 2012), may contribute to activation of protein degradation pathways and muscle atrophy. In a condition of energetic deficit due to reduced production and increased consumption of ATP due to statin-induced hyperexcitability and hypercontraction, AMPK activation was found to be increased likely at the aim to compensate for the lack of ATP (Richter and Hargreaves, 2013). However, this compensative effect may be limited by the statin-induced inhibition of glucose uptake through the GLUT-4 reduced expression.

Despite the up-regulation of MuRF-1 suggests intracellular protein degradation of specific substrate targets (Hanai et al., 2007), we found a noteworthy increase of Notch-1 in the biopsies of statin-treated patients. The Notch gene encodes an evolutionarily conserved cell surface receptor that generates regulatory signals based on the interactions between neighboring cells. Notch-1 is known to be involved in the proliferation of quiescent satellite cells and then in the regenerative potential of injured skeletal muscle (Conboy and Rando, 2005). Several studies have shown that Notch signaling favors myoblast proliferation, and, when sufficient myoblasts are produced, Notch signaling needs to be switched off, by appropriate molecules, to allow myoblast differentiation (Buas and Kadesch, 2010). At this regard it has been demonstrated that PKC activity may have a role in Notch-1 signaling activation (Goulas et al., 2012; Tremmel et al., 2013; Shin et al., 2014). Thus, statins seems to interfere with pathways involved in satellite cells activation and regeneration process. Accordingly, it has been found that staurosporine, an unspecific PKC inhibitor, have an atrophic effect in skeletal muscle (Mele et al., 2014), supporting the role of PKC in muscle cell regeneration and remodeling (Madaro et al., 2011; Marrocco et al., 2014). In agreement, we found an increase of the expression of PGC1α, a transcriptional coactivator of mitochondrial biogenesis and oxidative metabolism critical in the maintenance of glucose, lipid, and energy homeostasis in muscle and other tissues (Hanai et al., 2007). This increase correlates with the increased expression of one of the key enzymes controlling the Krebs cycle, the isocitrate dehydrogenase 3 (IDH3), which is also involved in anabolic signals (Yang et al., 2014) and an increased activity of the mitochondrial marker, CS, which indicates increased mitochondrial density possibly linked to higher ATP requirement for cell survival and/or sustained contraction. The activation of genetically programmed patterns, due to expression of myogenic factors during satellite cell recruitment, may also justify increased expression of different other genes. However, the lack of MyoD expression modification and the absence of centronucleated fiber suggests failure of further differentiation and interruption of muscle repair due to still unknown reasons. As already observed (Goodman et al., 2015), changes of PGC-1α expression and mitochondrial function occur independently from MuRF-1 increase, which likely affects other target proteins (Mallinson et al., 2009; Foletta et al., 2011; Chaanine et al., 2012).

Thus, a complex series of events is responsible for statin effects on skeletal muscle. In the Elderly, thought to experience a reduction of regenerative capacity (Blau et al., 2015) as well as an impairment of functional muscle properties, including a reduction of chloride channel expression and activity (Pierno et al., 2014), an increased risk of myopathy due to statin therapy may occur. However, the present study did not allow to confirm such hypothesis. A modification of the ClC-1 expression, or an increase of PKC expression and activity was observed in most of the subjects examined, independently from their age. Our hypothesis is that an increased risk of myopathy may raise only in the presence of concomitant harmful events. For instance, drug interaction or impaired statin elimination may be a cause (Gazzerro et al., 2012). In addition, toxicity studies showed that statin therapy plus eccentric exercise stress enable muscle instability, triggering stronger activation of intracellular proteolytic cascades (Urso et al., 2005). At this regard it is known that the mechanical stress during exercise may aggravate ClC-1 function (van Lunteren et al., 2007; Camerino et al., 2014b; Cozzoli et al., 2014) and that the activation of Ubiquitin Ligase Complex specifically mediates the poly-ubiquitination and degradation of ClC-1 (Chen et al., 2015). At the light of these findings, it would be also important to verify if strenuous exercise and/or genetic mutations of ClC-1, may potentiate adverse effects of statins, as already observed for metabolic myopathies (Tay et al., 2008).

Previous studies underline the problem of statin intolerance, defined as the inability to tolerate a therapeutic dose due to side effects including muscle symptoms, headache, sleep disorders, dyspepsia, nausea, rash, alopecia, erectile dysfunction, gynecomastia, and/or arthritis which may lead to cessation of therapy. Muscle symptoms, ranging from mild myalgia to rhabdomyolysis, are major side effects and a leading cause of statin intolerance. Statin intolerance usually develops in the first 3–6 months, thus in patients on long-term statin therapy, other factors, such as the nocebo effect or Vitamin D deficiency, may contribute to myalgia. It is thus possible that Vitamin D deficiency may have exacerbated statin-induced myopathy (Banach et al., 2015; Patel et al., 2016). However, we measured genes involved in muscle phenotype determination, calcium homeostasis, muscle atrophy and regeneration, as well as glucose metabolism. All the parameters found to be altered by statin-induced damage are potential biomarkers useful for diagnosis and prevention of statin associated muscle symptoms (SAMS). Some of them may be more sensitive than CK, since they are measured directly in the muscle. Indeed, symptoms, as myalgia and/or myopathy, may occur in the absence of CK elevation. The major difficulty is the bioptic sample collection. The ClC-1 channel could be a key biomarker of SAMS and the examination of its expression could be an additive test, especially useful in case of an uncertain diagnosis. In case of ClC-1 alteration, statin therapy should be discontinued. The use of more biomarkers is warranted for diagnosis, as this allows to reduce possible errors. This can be important for clinicians who must decide whether or not continue statin therapy in patients presenting myalgia, so to avoid the risk of life-threatening rhabdomyolysis (Gluba-Brzozka et al., 2016; Muntean et al., 2017).

## Limitations and Strengths

This study gives important information for statin therapy in humans, however, it shows some limitations. Due to the difficulty in collecting human tissues samples, we analyzed a small size of population. Twenty-two individuals reporting myalgia were screened in the Department of Clinical and Experimental Medicine of the University of Messina, and ten were deemed suitable for inclusion into the study. An advantage of a single-center cohort is the homogeneity of cohort with reduced variation in genetic and environmental factors. Despite the small sample size, the uniformity of data obtained allow us to hypothesize that the ClC-1 channel is a biomarker of statin-induced side effects. Further studies are warranted to confirm this hypothesis in a larger population.

Another limitation is the quantity of the biological material available. Indeed, it was not enough neither to perform both the real-time PCR and WB quantifications in the same individual, nor to perform WB for all the genes measured with real time PCR. The measure of ClC-1 protein is further restrictive due to the necessity to isolate biological membranes thus requiring more material. Thus, samples were randomly selected to perform real-time PCR or WB, in order to reduce classification errors. The control group was properly chosen, among those subject showing no sign of neuromuscular alteration or myopathy. A control group including patients taking statins but not experiencing muscle symptoms is lacking, because there are no clinical reason to take a biopsy in these individuals. In addition, patients suffering from myopathy but not using statin have not been included, because of the variability of clinical characteristics. Myopathy, characterized by structural and functional alteration of muscle fibers, can be of different etiologies and the analysis of ClC-1 channel is lacking in most of these conditions. We have chosen to exclude these subjects because a direct or indirect involvement of ClC-1 has been demonstrated in hereditary conditions, such as in Duchenne Muscular Dystrophy (De Luca et al., 2003) or during muscle atrophy/wasting due to aging, cachexia and/or denervation (Pierno et al., 2007). We found a stringent parallelism between statin toxicity and impairment of chloride channel function and expression either in rats or in patients, as demonstrated by this study. However, caution is necessary, because individual factors (SNPs) can be at the basis of susceptibility of statin effect. This aspect will be evaluated in further studies.

## Conclusion

Here we gained new information regarding protein expression and function in muscle biopsies of statin-treated patients who experience myalgia and cramps. In all the examined patients, statin therapy induced a loss of ClC-1 channel in skeletal muscle, already after a short period of treatment, which likely contribute to hyperexcitability and muscle damage. Thus, the quantification of this parameter in muscle biopsies may help diagnosis and/or risk evaluation of myopathy. The take-home message which can be drawn here is to be careful with statin therapy in physiopathological situations in which ClC-1 function is already modified.

## Author Contributions

Research design: GC, OM, AT, and SP. Conducted experiments: EC, KM, AF, EB, and MM. Contributed reagents, materials, analysis tool: ADL and AT. Analysis and interpretation of data: GC, OM, EC, KM, AF, EB, MM, CR, DT, CC, MC, and SP. Writing manuscript: GC, OM, AT, and SP. Critically read the manuscript: J-FD and ADL.

## Conflict of Interest Statement

The authors declare that the research was conducted in the absence of any commercial or financial relationships that could be construed as a potential conflict of interest.
